# Development and Validation of the Health Literacy Assessment Instrument for Patients with Chronic Pain

**DOI:** 10.1155/2021/9342746

**Published:** 2021-12-14

**Authors:** Siqing Chen, Xingwei Zhang, Meijuan Cao, Bingyu Zhao, Jie Fang

**Affiliations:** ^1^College of Humanities, Zhejiang Dongfang Polytechnic, Wenzhou 325000, Zhejiang, China; ^2^Division of Health Sciences, Hangzhou Normal University, Hangzhou 310036, Zhejiang, China; ^3^Affiliated Hospital of Hangzhou Normal University, School of Medicine, Hangzhou Normal University, No. 126, Wenzhou Road, Hangzhou 310036, Zhejiang, China

## Abstract

A suitable health literacy assessment instrument for patients with chronic pain (HLCP) in China with good instrument's psychometric properties is required. A theoretical framework for the HLCP was developed by adopting the hierarchical model of health literacy proposed by Nutbeam. The reliability and validity of the HLCP were tested in a cross-sectional survey of 237 chronic pain patients from three pain clinics and wards of Grade-3A hospitals in Zhejiang Province, China. The discriminant degree method, correlation analysis method, factor analysis method (exploratory factor analysis), half reliability, and other methods were utilized to screen items for inclusion in the final version of HLCP, and the fitness of the model was subsequently evaluated by confirmatory factor analysis. Cronbach's alpha value and test-retest with two-week intervals were used to test the internal consistency and retest reliability of the HLCP. In the exploratory factor analysis, three domains, functional health literacy (10 items), interactive health literacy (14 items), and critical health literacy (7 items), comprising 31 items in total, were finally loaded; the model was determined to explain 70.9% of the total variance. HLCP's effective assessment of the health literacy level of patients with chronic pain and its acceptable reliability and validity were revealed through the results.

## 1. Introduction

Chronic pain was declared a disease by the International Association for the Study of Pain in 2000 [1]. In many countries, the incidence of chronic pain among the general population is 20–45%; in China, there are over 300 million patients with chronic pain (21.43%), and this number increases by 20 million annually [2]. Chronic pain has been defined as persistent or recurrent pain that lasts for three months or more by the International Association for the Study of Pain [3]; however, the duration of pain in the definition has been shortened to one month by Chinese scholars, after considering the characteristics of patients with chronic pain [4]. Chronic pain can occur at any age and is persistent and recurrent; it also has a high incidence, affecting a large population, and a wide range of influence [5]. However, due to its complex etiology and difficulty in the diagnosis of chronic pain and as it does not directly endanger the patient's life, it can be easily ignored by researchers [6]. Nevertheless, abuse of analgesics is prominent worldwide [7, 8], and the impact of chronic pain on health problems and quality of life is attention worthy. For individuals with chronic pain, failure to obtain a timely diagnosis and reasonable treatment can lead to serious complications, which can not only cause further and possibly more intense pain but also lead to a heavy medical and economic burden to patients' families and society [9, 10].

The concept of health literacy was first proposed by Simonds in 1974 [11]; since then, explorations have been conducted in the field of health literacy by many researchers. Health literacy has been advocated by the World Health Organization and the American Medical Association, and an individual's capacity to obtain, process, and understand basic health information and the services needed to make appropriate health decisions have, thereby, been stipulated [12, 13]. The definition of health literacy proposed by the National Library of Medicine in 2000 is commonly cited [14]; interaction and criticism skills have been cited as important elements of health literacy in this definition. Following this, continuous improvements to the definition as well as an in-depth analysis of the connotations of health literacy have been sought by other organizations and well-known scholars, such as Nutbeam, a professor of public health at the Sydney School of Public Health. In China, research in this field commenced relatively late, with the concept of health literacy only being introduced in the country in 2005; nevertheless, the “Health China Initiative (2019–2030),” issued by the State Council, has listed “health knowledge popularization” as part of its first major action plan, which indicates that health literacy is being considered important in China.

With growing interest in health literacy, and as good health literacy is considered an important prerequisite to improve treatment outcomes of chronic pain, effective measurement of health literacy has become particularly important. Although there are many instruments that measure health literacy (both for China-based patients and patients in other countries) in the contexts of various common chronic diseases (e.g., hypertension, diabetes, stroke disease, chronic obstructive pulmonary disease, and tumors) [15–19], health literacy assessment instruments specialized for patients with chronic pain are lacking. Therefore, the development of a health literacy assessment instrument for patients with chronic pain (hereafter referred to as the “Health Literacy Assessment Instrument for Patients with Chronic Pain” (HLCP)) is warranted. Important reference data regarding the health literacy of this specific population could be provided by such an instrument, which could inform future measurements, intervention research, and practice.

## 2. Methods

### 2.1. Theoretical Framework

The theoretical framework used by the present study's authors to conceptualize the HLCP was based on Nutbeam's (2008) framework, which defines health literacy as comprising three dimensions: functional health literacy, interactive health literacy, and critical health literacy [20]. The characteristics of patients with chronic pain (obtained through discussions with patients) and the recommendations of relevant experts are combined in this study to define the dimensions and connotations of chronic pain. In the context of pain, functional health literacy refers to possessing basic reading, writing, and calculation skills required to obtain health information regarding pain. Possessing the knowledge to avoid the risks associated with chronic pain and improving one's knowledge of chronic pain, as much as possible, is the goal of functional health literacy. Next, interactive health literacy in this context refers to the ability to actively acquire information regarding chronic pain and apply it in order to change one's experience of pain. This element of health literacy concerns not only knowledge of chronic pain but also communication skills, problem-solving ability, and the ability to make health decisions. Finally, critical health literacy, in the context of pain, refers to the ability to critically analyze chronic pain based on one's own situation by using critical thinking. As this framework possesses a comprehensive interpretation of the definition and connotations of health literacy, this theory was suitable to determine the dimensions of the HLCP.

Behavioral change in humans can be divided into three interrelated processes: acquiring knowledge, generating belief, and forming behavior; this aligns with the Knowledge-Attitude-Behavior theory by Professor Mayo, which comprises knowledge, attitude, and behavior, and is associated with using health literacy to change one's health behaviors [21]. Jordan's health literacy theory involves studying the factors that affect access, understanding, and utilization of health information and health services from the perspective of patients. However, the following seven factors have been found to reflect the health literacy level at the individual level [22]. While the core contents of the three abovementioned theories are consistent, the highest level of cognition and skills (critical health literacy) is not reflected in the KAB theory and Jordan's health literacy theory. Information can be applied to control life events and health status by individuals with this type of literacy. The essence of the definition and connotation of these theories are integrated to design items for the evaluation instrument and to ensure the completeness and scientificity of these items.

### 2.2. Item Creation

The items for the assessment instrument were developed by creating an item pool that was based on expert guidance, the definition of chronic-pain-related health literacy, the Nutbeam health literacy model, KAB theory (and other theories), and by referring to the “pain management program” published by the British Pain Society in 2010 and the “CDC Guideline for Prescribing Opioids for Chronic Pain-United States” published by the Centers for Disease Control and Prevention in 2016 [23, 24]. First, the advantages and disadvantages of a variety of universal appraisal instruments were compared and analyzed. Further, items from existing health literacy assessment instruments [25–33], such as the Health Literacy Scale for Patients with Chronic Diseases, which is a scale that is based on Jordan's theory and was translated and adapted by Sun et al. in 2010 using data from interviews with patients with chronic diseases, were referred to [32]. Moreover, the actual situation of patients with chronic pain, including many influencing factors such as culture, economy, language, and medical system, was considered.

A group of experts was established, comprising five physicians specializing in pain, one head nurse of a pain department, two chief physicians, and two nursing professors. The inclusion criteria for this group were as follows: working in the field of pain medicine and health management for over 10 years, familiarity with providing health education to and communicating with patients, and holding an intermediate or higher professional title. Review, discussion, and analysis of the preliminary items were performed by the expert group.

The content validity of the framework was evaluated through expert consultations based on the Delphi method. Nineteen experts (in the areas of pain, health education, public health, nursing management, and/or health management) were invited to participate in two-round mail/e-mail consultations (from August to October 2020). They were asked to consider whether the chosen items represented actual situations encountered by patients with chronic pain and whether the items clearly described such situations. Several items were deleted or merged to create a list of 41 initial items based on expert recommendations and repeated discussions regarding the items among research team members. Of the original item pool, 40 items were modified to avoid ambiguity, 10 items were deemed irrelevant and removed, three new items were added, and the order of the individual items was adjusted. HLCP items are scored using a five-point Likert scale (5 = “strongly agree,” 4 = “agree,” 3 = “uncertain,” 2 = “disagree,” 1 = “totally disagree”), and the total is determined by summing the scores for each item. The higher the score, the higher the health literacy level of the respondent.

To determine the appropriate level of difficulty of the items, as well as their clarity and accuracy, a pre-test was performed, in which 15 patients with chronic pain were selected and administered the prototype instrument in the pain inpatient ward of a Hangzhou Grade-A hospital. The results showed that all patients could complete the questionnaire satisfactorily and could understand the meaning of each item. The average time for questionnaire completion was 10 minutes. The 41 items in this questionnaire were used to create the initial HLCP.

### 2.3. Design and Participants

To test the HLCP, 250 participants were aimed to be recruited between November 2020 and January 2021. According to the sample estimation method proposed by Kendall, the sample size is determined as 5 to 10 times the item of “Initial HLCP for the Health Literacy of Patients with Chronic Pain,” with a sample loss rate of 5–10%. The approximate sample size for this survey is 205–410; 250 questionnaires were actually distributed, and a random-sampling method was used. The participants were recruited from the pain clinics and wards of three Grade-A hospitals in Hangzhou, Zhejiang Province, China.

The inclusion criteria for the participants were patients with chronic pain, having a pain duration of over one month, being aged ≥18 years old, having normal thinking and language ability, having the ability to communicate orally and in writing, having clear consciousness, and having the ability to complete the scale. Participants with acute and serious illnesses, such as severe heart failure or advanced tumors, were excluded from the study. A total of 250 questionnaires were dispatched, along with an informed consent statement; the return of the questionnaire, which was completely voluntary and anonymous, was deemed to indicate consent. The questionnaire was in Chinese and translated into English by two professional researchers. The final sample size was 237 (response rate: 94.8%). The protocol of the present research was approved by the Ethics Committee of Hangzhou Normal University (20190090).

### 2.4. Statistical Analysis

The descriptive statistics were calculated (using frequencies and proportions) for the respondents' demographic data, which provided preliminary statistical information. Content validity was measured to determine whether the instrument content was capable of measuring the defined objective. Content validity refers to the consistency between the items in the assessment instrument and the content to be measured. To perform this, the opinion of five experts from the fields of pain, nursing management, and health management was sought. The experts were asked to evaluate each item in the instrument using a four-point Likert scale (1 = “unrelated,” 2 = “weak correlation,” 3 = “correlated,” 4 = “strong correlation”) and also to consider the scoring method and provide feedback. Based on these analyses, the item content validity index (I-CVI) and scale-content validity index (S-CVI) were measured. For the I-CVI and S-CVI, the criteria of item relevance, clarity, and simplicity were assessed, and for both the I-CVI and S-CVI, values >0.7 were considered acceptable.

Structural validity refers to the degree of agreement between the structure of the scale being tested and the theoretical structure [34]. The sample was randomly split for exploratory factor analysis (EFA; *n* = 237) and confirmatory factor analysis (CFA; *n* = 216), respectively. EFA was used to assess the construct validity. All 31 items were analyzed using principal component analysis with varimax rotation, using a special value cut-off of >1. A factor loading value of ≥0.4 was regarded as indicating a significantly related item. The adequacy of the sample size was assessed using the Kaiser–Meyer–Olkin test (KMO). After these analyses, seven items were removed. The fitness of the model was examined using the following series of indices: chi-square/degrees of freedom (*χ*^2^/df; threshold used to indicate a good fit: <3), root mean square error of approximation (RMSEA < 0.08), comparative fit index (CFI > 0.90), and Tucker–Lewis index (TLI > 0.90).

Reliability refers to the consistency and stability of the results produced by the instrument. Data for the total sample were used to examine the internal reliability (internal consistency) and external reliability (test-retest, split-half reliability) of the scale. Cronbach's *α* was used to assess the internal reliability of the scale. Based on suggestions made by previous researchers [35], Cronbach's *α* value of >0.7 was considered to be satisfactory. Then, to determine the external reliability, a retest was performed. The sample size for the test-retest was 1/10 of the original sample [14]. Thus, of the 237 patients who had initially completed the questionnaire, 24 patients with chronic pain were randomly selected from the total sample. These patients were retested every two weeks. Next, the intraclass correlation coefficient of the scores was calculated and used as the reliability coefficient. Correlation coefficients of 0.30–0.80 and *P* < 0.05 were considered acceptable.

Amos V.23.0 software and SPSS V.23.0 software were used for CFA and the aforementioned analyses, respectively.

## 3. Results

### 3.1. Respondents' Characteristics

A total of 237 participants included 91 males and 146 females; the male-to-female ratio was 1 : 1.60. The mean age was 45.9 (±15.3) years. Regarding ethnicity, the sample was mainly Han, which accounted for 99.2%; ethnic minorities accounted for 0.8%. Most participants were married (87.3%) followed by unmarried (6.3%), divorced (5.5%), and widowed (0.8%). Most participants resided in urban areas (55.3%) followed by rural areas (13.1%) and villages (31.7%). Regarding occupation, freelancer and professional or technical personnel were the most common occupations, accounting for 33.8% and 18.6%, respectively. The education level was mainly high school or vocational school, accounting for 43.9%. Regarding monthly income, 5,000–9,990 yuan and more than 10,000 yuan were most common, accounting for 33.3% and 41.4%, respectively. Medical expenses were met by most participants through medical insurance, (81.4%); self-funded and public expenses accounted for 9.7% and 8.9%, respectively (Table 1).

### 3.2. Content Validity of the HLCP

The I-CVI of the HLCP was 0.75–1.00, and the S-CVI was 0.95. This indicated that the content validity of the HLCP was confirmed by the expert panel.

### 3.3. Construct Validity of HLCP

A KMO statistic value of 0.95 was obtained, and Bartlett's test of sphericity was significant (*P* < 0.001); this indicated that the factor structure could be used for factor analysis. In EFA, using principal component analysis with varimax rotation, three domains with factor loadings of >50% were clearly distinguished (Table 2). Considering the theoretical structure of health literacy, these three domains were named “functional health literacy” (10 items), “interactive health literacy” (14 items), and “critical health literacy” (seven items), respectively. Seven items (Q13, Q16, Q24, Q27, Q30, Q34, and Q36) were removed due to low factor loadings (≤0.40) or serious cross-loadings. The final questionnaire consequently comprised 31 items. The three domains mentioned above explained 70.9% of the total variance observed. A good model fit of the three-component structure of the HLCP scale (31 items) was confirmed by the CFA; Further, the *χ*^2^/degrees of freedom = 2.182, the root mean square error of approximation = 0.074, the comparative fit index = 0.932, the Tucker–Lewis index = 0.922, the goodness-of-fit index = 0.801, the incremental fit index = 0.933, and the normed fit index = 0.883. All 31 items had factor loadings exceeding 0.4 (Table 2).

### 3.4. Reliability of the HLCP

Cronbach's *α* of the full scale was 0.97, and Cronbach's *α* values for each dimension ranged from 0.93 to 0.97. The test-retest reliability was 0.93. The split-half reliability of the full scale was 0.91 (Table 3). The reliability assessment of the questionnaire performed using Pearson's correlation also returned acceptable values (all values were between 0.61 and 0.83).

## 4. Discussion

To date, health literacy in regard to chronic pain has been explored by a variety of studies [25, 28, 31, 36–42]; however, they all relied upon universal health literacy instruments. Moreover, respondents' basic ability to read health information has been the focus of these health literacy assessment instruments; however, different domains such as interactive health literacy or critical health literacy in regard to health information processing and associated decision-making were not considered. Thus, a comprehensive assessment of the health literacy of patients with chronic pain has not been possible. Nevertheless, great variance in the health literacy levels of patients with chronic pain has been found in these studies.

The present study aimed to develop and assess the psychometric properties of the HLCP, which is designed to measure the health literacy of patients with chronic pain in China. The validity of the questionnaire was assessed using a factor analysis, which identified three domains. The contexts of these three domains, including functional health literacy, interactive health literacy, and critical health literacy, were consistent with the theoretical foundation and structure used to develop the health literacy scale. These three domains explained 70.9% of the cumulative variance observed in the results. The EFA performed in this study returned a KMO value of >0.8 and a cumulative variance contribution rate of ≥40%, and the factor loading of each item was >0.4. The common factors extracted from the scale were consistent with the theoretical framework, and the contents of the items within the three common factors were consistent with the theoretical basis and the health literacy framework for patients with chronic pain. The CFA was performed using chi-square statistics (*χ*^2^), RMSEA, GFI, NFI, INI, CFI, etc., as statistical indicators. It is generally recommended that an RMSEA value of <0.08 (the smaller the better) and NFI, TLI, INI, and CFI values of >0.9 indicate good model fit [34]. The NFI, TLI, and CFI of the three-factor model all exceeded 0.9, and the RMSEA value was 0.074, which indicate that the three-factor model had a good overall fit. The model fit in the present study was better than that of the model developed by Haolin et al. [32]. A health literacy scale for patients in China with chronic diseases, HLSC, was developed by Haolin et al. and 67.5% of the cumulative variance could be explained by their model.

The presence of content validity in the HLCP was agreed upon by the panel of experts. Cronbach's *α* for the full scale was 0.97, and Cronbach's *α* for the subscales ranged from 0.93 to 0.97, verifying the scale's internal consistency. Test-retest assessments revealed that the questionnaire was reliable and could be administered at different times and locations. The internal consistency coefficient and test-retest reliability coefficient indicated that the HLCP had great reliability and stability. Thus, HLCP's ability to meet or exceed psychometric standards was revealed in this study's findings.

Similarly, the scale by Haolin et al. showed an internal consistency of 0.89, while the internal consistency among the subscales ranged from 0.86 to 0.94 [32], Further, an instrument for measuring health literacy among patients with chronic diseases, developed by Leung, had an internal consistency of 0.91. Further, a Chinese health literacy scale for patients with diabetes was also developed by Leung et al. The internal consistency and the consistency of its four subscales were 0.884, 0.885, 0.667, 0.654, and 0.717, respectively [18, 43].

The characteristics of patients with chronic pain were taken into account by the HLCP; moreover, multiple dimensions of the health literacy of such patients were measured. First, functional health literacy is reflected in items 1–7, focusing on the patient's knowledge of the specifics of chronic pain. In the functional health literacy dimension, the items were based on the basic knowledge of chronic pain, the onset characteristics, development trends and prognoses, prevention and treatment-related strategies, pain medication, etc. Second, negative psychological conditions, e.g., distress and depression, are reflected in item 8 of this assessment instrument, as these conditions are often experienced by patients with chronic pain. The above items were designed to determine the patient's basic knowledge of pain. In addition, the patient's understanding of pain treatment is measured through item 4. Next, management of chronic pain through exercises, relaxation therapy, and nondrug therapy should be understood by patients with chronic pain, so that they can better manage their own pain. Therefore, pain management guidelines were reflected in items 9 and 10. Third, patients with chronic pain must be capable of reading and writing relevant chronic pain information and calculating pain medication dosage; the relevant content was incorporated into items 4, 5, 6, and 7.

Measurement of patients' functional health literacy as well as interactive and critical health literacy was the focus of this instrument. The influencing factors of health literacy included not only an individual's education level and disease status but also the economic status and social support, among other factors. Therefore, the evaluation instrument must be able to measure these influencing factors. Accessibility of medical resources is defined through economic status as limited health behaviors are adopted by people with high health literacy and limited funds; this was reflected in items 16 and 17.

Regarding interactive health literacy, seeking such information in a proactive manner and using a range of approaches to obtain it were effectively measured by items 20, 21, and 23. Further, the measurement of the patient's ability to make health decisions and the patient's problem-solving ability was seen in items 18–22 and items 21 and 24, respectively. The patient's information acquisition ability is reflected in these two abilities (i.e., whether the patient actively seeks to obtain pain information and applies the new knowledge obtained to change his/her own health status). Additionally, the key elements of interactive health literacy emphasized in the definition of interactive health literacy include the ability to communicate, solve problems, and make decisions to treat diseases. The patient's belief in their ability to improve their chronic pain and willingness to do so are measured in items 11–14, 20, and 21.

Regarding critical health literacy, the definition of this type of health literacy is reflected in item 28, as patients' application of critical thinking to analyze chronic pain information was examined through it. Critical thinking must be used by patients to analyze information concerning chronic pain in the contexts of their actual situations; this is reflected in items 25–30.

First, the common characteristics of patients with chronic pain and the actual situations of such patients are considered in the HLCP. Patients with chronic pain who have low health literacy are more likely to have inaccurate knowledge regarding medication, and the risks associated with such a lack of information are often underestimated. For example, while the abuse of analgesics is prominent in the US [44, 45], the fear of painkiller side effects, dislike for painkillers, and the belief that they are not helpful for relieving pain are common among patients with chronic pain in China [46]. Second, chronic pain and depression have the same neurobiological mechanism, and patients with chronic pain are relatively likely to develop symptoms of depression [47, 48]; thus, patients should be aware of this risk. Third, medication concepts, as well as pain beliefs, have an important impact on health literacy. Fourth, improvement in health literacy is associated with better pain self-management among patients with chronic pain [49]. Thus, health literacy is important for patients with chronic pain due to several reasons.

Compared with existing instruments for assessing health literacy among patients with chronic disease, the characteristics of chronic pain are better reflected in the HLCP. Moreover, the health literacy level of patients with chronic pain is accurately, scientifically, and comprehensively assessed through it. Therefore, the HLCP is considered more appropriate for use among patients in China compared with the existing health literacy scales for chronic patients, including HLSC and universal health literacy instruments. Data obtained through the HLCP, which accounts for the multiple dimensions of health literacy (functional health literacy, interactive health literacy, and critical health literacy), can provide insights for future research on health literacy.

Several suggestions can be made based on the findings of this study. First, there is a growing interest in exploring the factors influencing the health literacy of patients with chronic pain. However, to date, existing research and instrument development in this regard have been insufficient. Thus, future studies should draw attention to the above aspect. Second, little evidence exists regarding the influence of health literacy on chronic pain outcomes. The relationship between pain outcomes and health literacy should be explored in future studies. Third, after using the HLCP to measure the health literacy (functional, interactive, and critical health literacy) of patients with chronic pain in China, the health literacy of such patients can be improved through interventions implemented at the individual, family, hospital, community, and government level. Health outcomes, prognoses, and quality of life could be improved, and the goal of good national health could be achieved.

## 5. Conclusion and Limitations

In conclusion, considering the high validity and reliability of the 31-item HLCP, as well as its advantages, such as the relatively low number of questions, its ease of implementation, and its ability to assess health literacy among patients with various kinds of chronic pain, the HLCP appears to be an appropriate instrument for assessing the health literacy of patients with chronic pain in China and has wide application value.

This study has some limitations. First, as a result of restrictions regarding time and funding, all samples recruited were from Zhejiang Province; this may have generated selection bias. Second, some of the health information used in the HLCP is context-specific. To administer the HLCP in other regions of China, some items needed to be reexamined to ensure that they are suitable for the contexts in question. Third, although factors common to a variety of chronic pain diseases are focused on in the HLCP, it might not be suitable for certain kinds of chronic pain that have unique characteristics. Although the instrument developed and validated in this study offers a sound basis, further studies with larger sample sizes are required. In-depth exploration of patients with chronic pain is needed to continuously verify the reliability and validity of the evaluation instrument.

## Figures and Tables

**Table 1:** Respondents' characteristics.

Variable	*N*	%
*Sex*		
Male	91	38.4
Female	146	61.6

*Age (years)*		
20–30	22	9.3
31–40	40	16.9
41–50	73	30.8
51–60	79	33.3
>60	23	9.7

*Ethnicity*		
Han Chinese	235	99.2
Minority	2	0.8

*Marital status*		
Unmarried	15	6.3
Married	207	87.3
Widowed	2	0.8
Divorced	13	5.5

*Location*		
Rural area	31	13.1
Village	75	31.7
Urban area	131	55.3

*Occupation*		
Laborer	14	5.9
Farmer	13	5.5
Government worker	21	8.9
Professional or technical personnel	44	18.6
Freelancer	80	33.8
Service personnel	4	1.7
Commercial worker	5	2.1
Unemployed	41	17.3
Retiree	15	6.3

*Education level*		
Junior middle school or below	36	15.2
High school or vocational school	104	43.9
Technical/junior college	39	16.6
Bachelor's degree or higher	59	24.9

*Household income (RMB per month)*		
0–999	7	3.0
1,000–2,999	13	5.5
3,000–4,000	40	16.9
5,000–9,990	79	33.3
≥10,000	98	41.4

*Method of paying medical costs*		
Health insurance	193	81.4
Self-payment	23	9.7
Public expense	21	8.9

**Table 2:** Results of exploratory factor analysis with varimax rotation.

No.	Items	Dimension 1 (functional health literacy)	Dimension 2 (interactive health literacy)	Dimension 3 (critical health literacy)
1	I have knowledge of the anatomy and physiology of pain, as well as its mechanism of conduction	0.806		
2	I know the difference between acute pain and chronic pain	0.804		
3	I am knowledgeable regarding the classification and nature of chronic pain	0.876		
4	I am familiar with essential information concerning chronic pain, such as the working hours and locations of pain specialists	0.727		
5	I have knowledge of strategies and solutions for preventing and treating chronic pain and do not have difficulties acquiring relevant information	0.877		
6	I have knowledge of the characteristics, developmental trends, and prognoses of chronic pain	0.895		
7	I have knowledge of the nature, dosage instructions, and side effects of analgesic medicine and can complete related medical forms calculating the pain medication dose when necessary	0.872		
8	I have knowledge of the main comorbidities of chronic pain, such as distress and depression, and many other psychological issues	0.867		
9	I have knowledge of the safety and risks of increasing my engagement in activities	0.810		
10	I have knowledge of strategies and relaxation techniques for improving sleep	0.679		
11	I am willing to accept therapy for chronic pain		0.774	
12	I trust physicians and therapeutic methods		0.765	
13	I have confidence in my self-care abilities and the effects of therapy for chronic pain		0.735	
14	I am willing to emphasize my needs regarding physiological, psychological, and social support for chronic pain		0.764	
15	I am willing to spend time and effort to relieve my pain		0.793	
16	I am willing to pay for my medical expenses (mainly referring to out-of-pocket expenses)		0.806	
17	I am willing to pay for chronic pain management (referring to medical costs)		0.808	
18	I adhere to physicians' advice		0.770	
19	I make some preparations before visiting the physician		0.600	
20	I engage in adequate communication with health-care providers regarding the status of my pain		0.687	
21	I proactively seek help when I do not comprehend something relating to the prevention and control of chronic pain		0.462	
22	I implement useful information I have obtained regarding chronic pain prevention and control		0.595	
23	I make detailed inquiries when I do not understand physicians' explanations		0.590	
24	I perform timely identification of my own chronic pain and seek professional help		0.557	
25	When chronic pain occurs, I can implement coping strategies in a timely manner			0.631
26	I can use analgesic medical equipment/instruments to relieve chronic pain			0.515
27	I can adjust my personal mental outlook to manage the psychological outcomes of chronic pain			0.501
28	I can collect different opinions about health information and analyze and compare their applicability, accuracy, and reliability			0.622
29	I can self-monitor my pain			0.605
30	I can make decisions regarding analgesics by incorporating physicians' advice with the characteristics of my own situation			0.651
31	I can take an objective view of the adverse effects of analgesic drugs and proactively address these effects			0.547

Dimension 1 (functional health literacy) = possessing basic reading, writing, and calculation skills required to obtain health information regarding pain. Dimension 2 (interactive health literacy) = the ability to actively acquire and apply chronic pain information in daily life to change pain conditions. of pain. Dimension 3 (critical health literacy) = the ability to critically analyze chronic pain based on one's own situation by using critical thinking.

**Table 3:** Cronbach's *α* and intraclass correlation coefficient for the HLCP and its subscales.

Domains	Number of items	Cronbach's *α*	Intraclass correlation coefficient
Functional health literacy	10	0.97	0.74
Interactive health literacy	14	0.95	0.60
Critical health literacy	7	0.93	0.67

## Data Availability

The data used to support the findings of this study are included within the article.
